# Machine Learning-Assisted Measurement Device-Independent Quantum Key Distribution on Reference Frame Calibration

**DOI:** 10.3390/e23101242

**Published:** 2021-09-24

**Authors:** Sihao Zhang, Jingyang Liu, Guigen Zeng, Chunhui Zhang, Xingyu Zhou, Qin Wang

**Affiliations:** 1 Institute of Quantum Information and Technology, Nanjing University of Posts and Telecommunications, Nanjing 210003, China; 1018010426@njupt.edu.cn (S.Z.); 1018010421@njupt.edu.cn (J.L.); zgg@njupt.edu.cn (G.Z.); chz@njupt.edu.cn (C.Z.); 2 Broadband Wireless Communication and Sensor Network Technology, Key Lab of Ministry of Education, Nanjing 210003, China; 3 Telecommunication and Networks, National Engineering Research Center, NUPT, Nanjing 210003, China

**Keywords:** measurement-device-independent quantum key distribution, reference frame calibration, machine learning, transmission efficiency, biased basis choice

## Abstract

In most of the realistic measurement device-independent quantum key distribution (MDI-QKD) systems, efficient, real-time feedback controls are required to maintain system stability when facing disturbance from either external environment or imperfect internal components. Traditionally, people either use a “scanning-and-transmitting” program or insert an extra device to make a phase reference frame calibration for a stable high-visibility interference, resulting in higher system complexity and lower transmission efficiency. In this work, we build a machine learning-assisted MDI-QKD system, where a machine learning model—the long short-term memory (LSTM) network—is for the first time to apply onto the MDI-QKD system for reference frame calibrations. In this machine learning-assisted MDI-QKD system, one can predict out the phase drift between the two users in advance, and actively perform real-time phase compensations, dramatically increasing the key transmission efficiency. Furthermore, we carry out corresponding experimental demonstration over 100 km and 250 km commercial standard single-mode fibers, verifying the effectiveness of the approach.

## 1. Introduction

Based on the laws of quantum physics [1], quantum key distribution (QKD) can in principle provide unconditional security between two legitimate users (Alice and Bob) [2]. However, due to the loopholes of imperfect devices, the security of practical QKD systems are vulnerable to various attacks by an evil eavesdropper (Eve) [3,4,5]. In order to resist the attacks, plenty of methods have been proposed, such as the decoy-state method [6,7], the measurement device-independent QKD (MDI-QKD) [8]. Combined with the decoy-state method, MDI-QKD can resist the loopholes from detector side-channel attacks and multi-photon components in sources, and thus has attracted extensive attention [9,10,11,12,13,14,15].

Meanwhile, how to implement the reference frame calibration is a significant challenge. Because of the phase fluctuation, the reference frame needs to be calibrated in a timely manner. Previously, an original approach is using scanning-and-transmitting program to calibrate the phase drift. Bob scans his phase modulation voltage while Alice fixes her phase voltage to ascertain the zero-phase voltage of the minimum count. Two users process feedback control according to the fitted zero-phase voltage. Consuming extra calibration time, the scheme keeps the stability of the QKD system at the cost of the so-called duty cycle [16], which refers to the ratio of the transmission time to the total time. Another widely used scheme is inserting a phase stabilization laser (PSL) which has the same wavelength as the signal laser’s between the interferometers of two users with an additional fiber link, which increases the system complexity. In the network [17], the PSL and an extra interferometer are employed in the relay to calibrate the reference frame between each two users, increasing the system complexity. Recently, data-driven machine learning based on complex data analysis methods for quantum control have been proposed [18,19,20]. In order to overcome those shortcomings, we adopt long short-term memory network (LSTM) [21,22] onto the MDI-QKD system and predict out the phase drift between two users in advance. Immediately, real-time phase compensations can be realized, drastically increasing the key transmission efficiency [23].

This paper is arranged as follows. In Section 2, we introduce the details about our machine learning model, and in Section 3 we tell how to run it on the MDI-QKD system with biased base choice. In Section 4, experimental data are analyzed and discussed. Finally, summaries and outlooks are given in Section 5.

## 2. Methods

**Figure 1:**
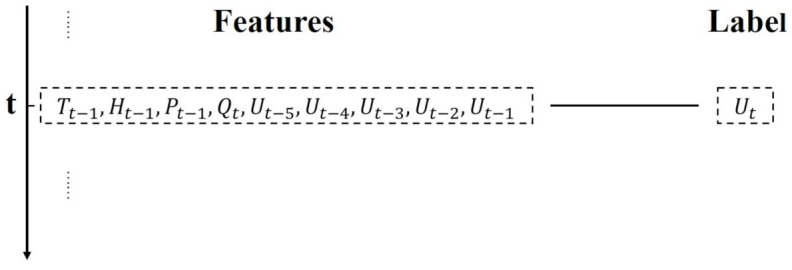
Data structure of *t* moment. T, temperature; H, humidity; P, intensity of laser; Q, QBER of XX basis-pair; U, voltage.

The inevitable change of arm length difference in interferometers of Alice and Bob leads to the phase drift, which introduces further errors in *X* basis. Adopting machine learning technique to efficiently predict the phase drift instead of using the time-consuming scanning-and-transmitting program continually. We conduct supervised learning here to enable the LSTM network to extract phase drift information. A mass of data points should be collected for training the network before prediction, which is performed in advance. The whole data consists of the features and label of various time moments [24], including the operating temperature, the humidity, the intensity of a laser, the partially disclosed quantum bit error rate (QBER) of XX basis-pair, and five time-series displacement voltages, which can be obtained by running the MDI-QKD system with the traditional scanning-and-transmitting program. Moreover, the label is zero-phase voltage of the next moment. The data structure is illustrated in Figure 1. A newly added data feature, i.e., partially disclosed QBER of XX basis, can provide the LSTM network valuable running status, which directly increase prediction accuracy compared to the former data features [23]. The training data conclude 25 batches, and each batch consists of 5400 data points. The testing data conclude 3 batches and each batch has the same number of data points as the training data. Apart from that, we randomly extract one batch from training data and one batch from test data for cross validation check.

**Figure 2:**
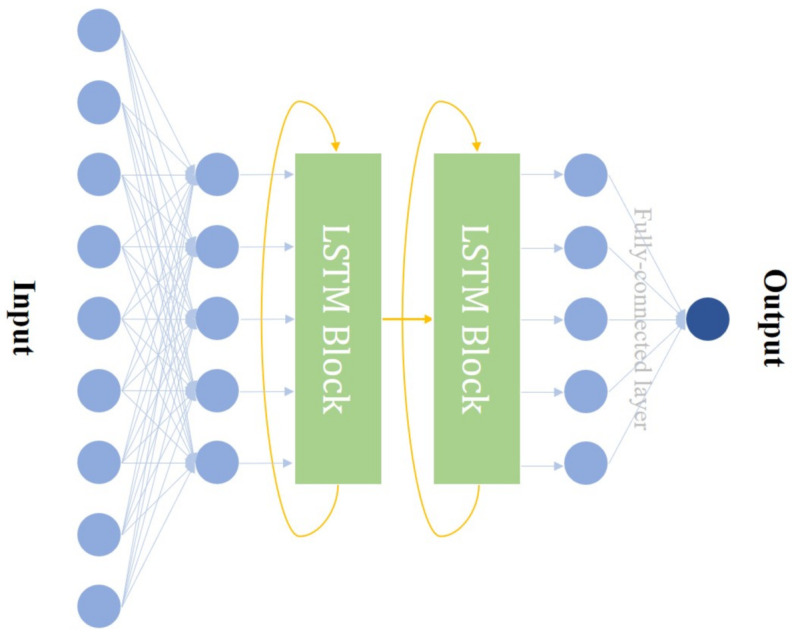
Diagram of the two-layer LSTM network.

Here, we design a two-layer LSTM network, which is illustrated in Figure 2, and utilize a mean squared error (MSE) cost function. This two-layer LSTM structure can extract temporal information from both coarse and fine granularity, which will help improve prediction accuracy, and detailed comparisons of different models are given in Appendix A. The number of hidden neurons in the first layer is 14 and the number of hidden neurons in the second layer is 9, which is obtained by prune. An unified Max-min normalization has been adopted for all data before input into the network. After the normalization, a two-layer feedforward network with 9 neurons and 5 neurons is placed before the LSTM as an encoder for feature extraction and data denoising. The output of the second LSTM layer is input into a fully-connected layer, which decodes the output into the zero-phase voltage of the next moment and applies it to Bob’s phase modulator (PM). We use Adam as the optimization algorithm for 350 epochs, which takes approximately 40 min on our PC (CPU: Intel Core i7 9700@ 3.6 GHz; GPU: NVIDIA GeForce RTX 2080; RAM: DDR4 8 GBytes). The initial learning rate is 0.025, and it drops 70% every 100 epochs. Batch training is adopted. The final MSEs of training set, testing set, and validation set are 0.0533, 0.1131, and 0.881 respectively.

Additionally, an updating process is added periodically for the long-term reliability of network forecasting, in which the scanning program will be operated after predicting for a certain time to eliminate the cumulative error in the prediction period [23]. As a result, the duty ratio of the present MDI-QKD system has been increased from 85.7% (transmitting: 30 s, scanning: 5 s) to 96.9% (transmitting: 540 s, mismatch events: 2 s, updating: 15 s).

## 3. Experiment

MDI-QKD achieves a better balance between security and practicality, while it generates lower secret key rate than BB84 protocol, especially considering the finite data size effect. For the sake of improving the performance, different approaches and strategies have been proposed and experimentally verified [14,25,26,27,28]. Here, we investigate the implementation of LSTM model based on a simple three-intensity decoy-state MDI-QKD scheme with biased basis choice [15], in which the decoy pulses are only prepared in X basis.

In the biased three-intensity decoy-state MDI-QKD protocol, first, Alice and Bob randomly prepare phase-randomized weak coherent state (WCS) pulses into three different intensities (*u*,*v*,*o*) with certain probabilities, each corresponds to the intensity of the signal state, the decoy state, and the vacuum state respectively. The signal pulses are prepared either in *Z* or *X* basis. Different from standard three-intensity decoy-state schemes [25], the decoy pulses are prepared only in *X* basis. Then, Charlie performs Bell-state measurements on the pulse pairs from both Alice and Bob and announces the results of the effective events. Finally, Alice and Bob exchange the basis-choice information and carry out parameter estimation and postprocessing processes. Finally, the lower bound of key rate can be calculated as follows [14,15,25]:

(1) $$R⩾p_{\mu_{A}}p_{Z|\mu_{A}}p_{\mu_{B}}p_{Z|\mu_{B}}\left\{a_{1}^{\mu }b_{1}^{\mu }Y_{11}^{Z,L}\left[1-H_{2}\left(e_{11}^{X,U}\right)\right]-S_{\mu \mu }^{ZZ}fH_{2}\left(E_{\mu \mu }^{ZZ}\right)\right\}$$

where $S_{\mu \mu }^{ZZ}$ and $E_{\mu \mu }^{ZZ}$ each represents the average counting rate and the average QBER in Z basis; $Y_{11}^{Z,L}$ and $e_{11}^{X,U}$ each denotes the yield and the phase-flip error-rate of the single-photon-pair pulses in Z basis, in which the superscript *L* and *U* each represents the lower bound and upper bound, respectively; *f* is the inefficiency of the error correction and we reasonably assume *f* = 1.16; and $H_{2}\left(x\right)$ is the binary Shannon information function, defined as $H_{2}\left(x\right)=-xlog_{2}\left(x\right)-\left(1-x\right)log_{2}\left(1-x\right)$.

**Figure 3:**
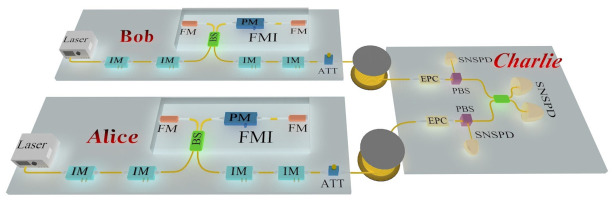
Schematic setup of MDI-QKD system. Laser, continuous-wave laser; IM, intensity modulator; PM, phase modulator; FM, Faraday mirror; ATT, attenuator; EPC, electronic polarization controller; BS: beam splitter; SNSPD, super-conducting nanowire single-photon detector.

The MDI-QKD experiment setup is demonstrated in Figure 3. We apply the time-bin phase encoding scheme, and utilize intensity modulators (IMs) and Faraday–Michelson interferometers (FMIs) [29] as the key apparatus for source encoding. The two legitimate users, Alice and Bob, which are symmetrical to an unreliable relay Charlie, each owns a narrow linewidth continuous-wave laser whose frequencies are locked to the molecular absorption line with a center wavelength of 1550.0 nm. The light sources, which generate continuous wave, are precisely chopped into pulse trains with a 3 ns temporal width and a repetition rate of 50 MHz by two IMs: the former is used for decoy-state modulation and the latter for extinction ratio improvement.

Next, Alice and Bob send their signal laser pulses to Charlie for a partial Bell state measurement with two super-conducting nanowire single-photon detectors (SNSPDs). The detectors operate at 2.2 K, providing a 80% detection efficiency at the dark count rate of 10 counts per second. The efficiency could still maintain 60% with the loss of devices at Charlie’s side, including an electric polarization controller (EPC), a polarization beam splitter (PBS), a beam splitter (BS), and detectors. The count results from two SNSPDs are recorded by a time-to-digital converter with 4 ns gate.

In order to realize the stable Hong–Ou–Mandel (HOM) interference, the indistinguishability of the signal pulses in spectrum, timing, and polarization must be guaranteed. Any difference in these dimensions will bring errors in the X basis. In our experiment, we utilize two narrow line width continuous-wave lasers with high accuracy in the frequency domain. Additionally, we apply an optical delay (OD) in Alice’s station to adjust the arriving time. For the polarization mode, we insert a polarization stabilization system, composed of an EPC, a PBS and a SNSPD before the interference. By monitoring the reflection counts from the PBSs with two SNSPDs, the EPCs could compensate for the polarization drifts every 30 min.

We run the machine learning-assisted MDI-QKD system in the laboratory with spooled fibers (0.18 dB/km) over 100 and 250 km, respectively. A total of $10^{12}$ pulses are sent from each user at different distances. We make the finite-key analysis and set the failure probability as $10^{-7}$[15]. With the decoy-state method, we apply the collective constraints and joint parameters estimation techniques to estimate $Y_{11}^{Z,L}$ and $e_{11}^{X,U}$ [15].

## 4. Discussion

**Figure 4:**
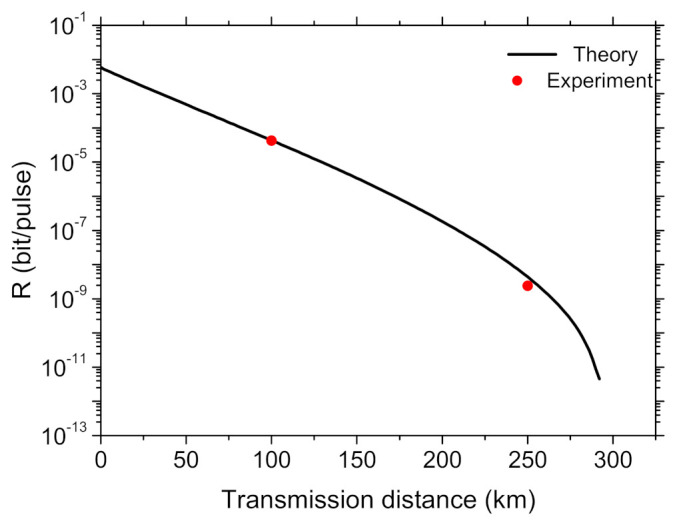
Theoretical and experimental results of the key rate’s dependence on the transmission distance. system parameters are as follows: dark counting rate per pulse and overall efficiency of detection side are $4\times 10^{-8}$ and 60%; misalignment errors in Z and X bases are 0.15% and 1.5%, respectively.

**Figure 5:**
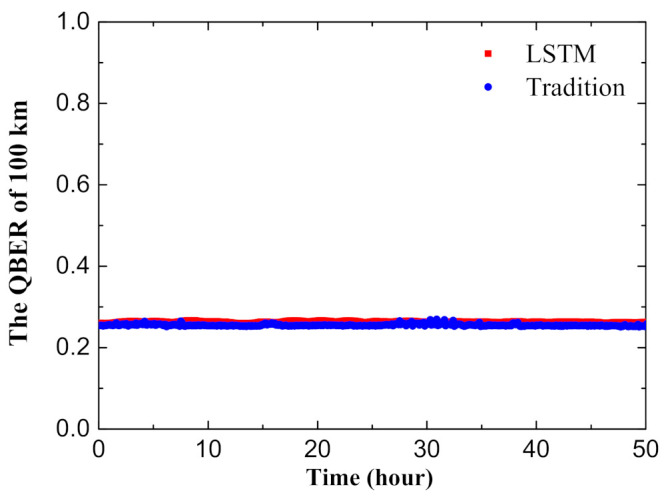
Comparisons between applying traditional scanning-and-transmitting program and using current LSTM-model-based QKD systems on QBER at the transmission distance of 100 km.

The theoretical simulations and experimental results are shown in Figure 4. With our current LSTM model-based method, we gain a good and similar result which agrees well with the theoretical predictions, due to the stability of the system and the updating and feedback data. With the data given in Table 1, Table 2, and some other experimental results, we can evaluate the phase error rates and the final secure key rates. At 100 km and 250 km, we obtain the key rates of $4.24\times 10^{-5}$ and $2.41\times 10^{-9}$ per pulse, individually. We also run the system with traditional scanning-and-transmitting method at the same environment for comparison. Note that we can get the similar level of key rate per pulse by using our present machine learning-assisted MDI-QKD system compared with using traditional scanning-and-transmitting mode. However, the duty cycle of our present method has been increased by more than 10 percent, giving a significant improved transmission efficiency. On the other hand, in order to verify the long time stability, we run the MDI-QKD system at the transmission distance of 100 km over 48 h and record corresponding QBER, using either machine learning-assisted mode or traditional scanning-and-transmitting mode, see Figure 5. Obviously, these two methods exhibit similar level of QBER and stability. In short, the new method not only improves systematic transmission efficiency, but also avoids potential vulnerabilities as no additional hardware is introduced. Furthermore, the duty cycle and stability time can be further improved by program optimization to match the requirements of various systems.

## 5. Conclusions

In conclusion, we developed a machine learning-assisted system, where the LSTM network is for the first time to implemented onto the MDI-QKD system for reference-frame calibrations. Furthermore, we carry out experimental demonstrations over 100 km and 250 km transmission distances by running the present MDI-QKD system. Experimental results show that our present machine learning-assisted mode can dramatically improve the transmission efficiency of MDI-QKD systems compared with using the traditional scanning-and-transmitting approach. Meanwhile, our present system can keep quite good stability over long time running. In addition, the biased basis choice idea has been employed to reduce the redundancy of decoy pulses in Z basis, and thus diminish the influence of the finite-data-size effect. Therefore, our present work can provide valuable references for the implementation of large-scale quantum communication [30,31] in the near future.

## Figures and Tables

**Table 1:** Optimized parameters of sources with 100 km and 250 km fibers.

Parameters	μ	*v*	ω	$P_{\mu }$	$P_{v}$	$P_{X|\mu }$
100 km	0.6353	0.0476	0.3171	0.885	0.110	0.002
250 km	0.4492	0.0691	0.4817	0.495	0.436	0.027

**Table 2:** Crucial values in the key rate formula: estimated single photon yield ($Y_{11}^{Z,L}$) and phase error rate ($e_{11}^{X,U}$); measured values of QBER ($E_{\mu \mu }^{ZZ}$, $E_{\mu \mu }^{XX}$) and gain ($Q_{\mu \mu }^{ZZ}$, $Q_{\mu \mu }^{XX}$) when Alice and Bob both prepare the signal state in Z basis, X basis.

Distance	$Y_{11}^{Z,L}$	$e_{11}^{X,U}$	$Q_{\mu \mu }^{\mathrm{ZZ}}$	$E_{\mu \mu }^{\mathrm{ZZ}}$	$Q_{\mu \mu }^{\mathrm{XX}}$	$E_{\mu \mu }^{\mathrm{XX}}$
100 km	0.0015	0.1539	$4.57\times 10^{-4}$	0.002	$9.16\times 10^{-4}$	0.257
250 km	$3.0182\times 10^{-6}$	0.3118	$5.597\times 10^{-7}$	0.00236	$1.085\times 10^{-6}$	0.267

**Figure A1:**
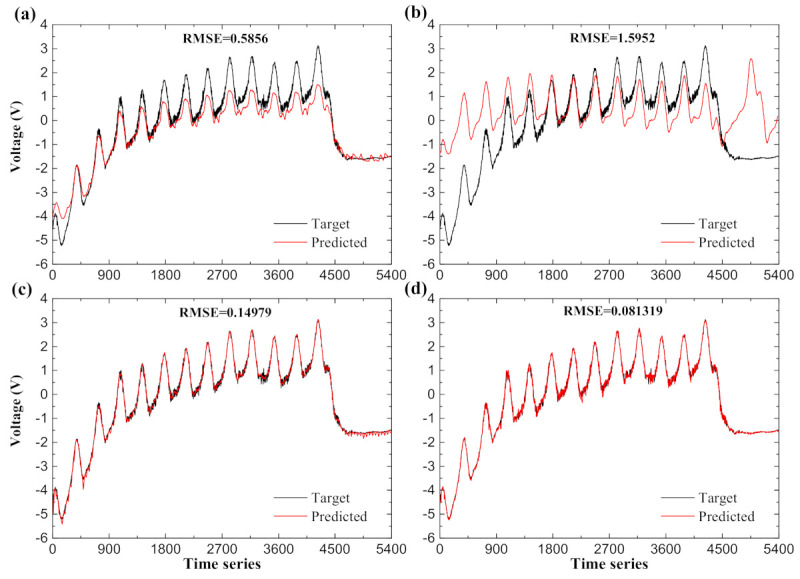
Comparisons of prediction results from (**a**) ARMA, (**b**) feed-forward neural network (one hidden layer with 50 neurons), (**c**) one-layer LSTM network, and (**d**) two-layer LSTM network.

## Appendix A

Here, we give comparisons among the predictions of different models, including autoregressive moving average model (ARMA), feed-forward neural network, one-layer LSTM network, and two-layer LSTM network. Taking a time series of 5400 testing data points for instance, the prediction results and their root mean square errors (RMSE) are illustrated in Figure A1. The feed-forward neural network is not a good choice for dealing with time series, and the result shows more of its fixed input–output mapping. Yet, the ARMA gives a more fitting prediction than it of feed-forward neural network. On the other hand, the two-layer LSTM network can give a better prediction compared to one-layer LSTM network especially on fine granularity.
